# Oligodendroglia-derived extracellular vesicles activate autophagy via LC3B/BAG3 to protect against oxidative stress with an enhanced effect for HSPB8 enriched vesicles

**DOI:** 10.1186/s12964-022-00863-x

**Published:** 2022-05-05

**Authors:** Bram Van den Broek, Charlotte Wuyts, Angela Sisto, Isabel Pintelon, Jean-Pierre Timmermans, Veerle Somers, Vincent Timmerman, Niels Hellings, Joy Irobi

**Affiliations:** 1 Department of Immunology and Infections, Biomedical Research Institute, Hasselt University, Hasselt, Belgium; 2 Peripheral Neuropathy Research Group, Department of Biomedical Sciences, Institute Born Bunge and University of Antwerp, Antwerp, Belgium; 3 Laboratory of Cell Biology & Histology, Antwerp Centre for Advanced Microscopy (ACAM), University of Antwerp, Antwerp, Belgium

**Keywords:** CNS, Engineered nanocarriers, Extracellular vesicles, Microglia, Molecular chaperones, Cellular inflammation, Oligodendroglia, Small heat shock protein 8

## Abstract

**Background:**

The contribution of native or modified oligodendroglia-derived extracellular vesicles (OL-EVs) in controlling chronic inflammation is poorly understood. In activated microglia, OL-EVs contribute to the removal of cytotoxic proteins following a proteotoxic stress. Intracellular small heat shock protein B8 (HSPB8) sustain this function by facilitating autophagy and protecting cells against oxidative stress mediated cell death. Therefore, secretion of HSPB8 in OL-EVs could be beneficial for neurons during chronic inflammation. However, how secreted HSPB8 contribute to cellular proteostasis remains to be elucidated.

**Methods:**

We produced oligodendroglia-derived EVs, either native (OL-EVs) or HSPB8 modified (OL-HSPB8-EVs), to investigate their effects in controlling chronic inflammation and cellular homeostasis. We analyzed the impact of both EV subsets on either a resting or activated microglial cell line and on primary mixed neural cell culture cells. Cells were activated by stimulating with either tumor necrosis factor-alpha and interleukin 1-beta or with phorbol-12-myristate-13-acetate.

**Results:**

We show that OL-EVs and modified OL-HSPB8-EVs are internalized by C20 microglia and by primary mixed neural cells. The cellular uptake of OL-HSPB8-EVs increases the endogenous HSPB8 mRNA expression. Consistently, our results revealed that both EV subsets maintained cellular homeostasis during chronic inflammation with an increase in the formation of autophagic vesicles. Both EV subsets conveyed LC3B-II and BAG3 autophagy markers with an enhanced effect observed for OL-HSPB8-EVs. Moreover, stimulation with either native or modified OL-HSPB8-EVs showed a significant reduction in ubiquitinated protein, reactive oxygen species and mitochondrial depolarization, with OL-HSPB8-EVs exhibiting a more protective effect. Both EV subsets did not induce cell death in the C20 microglia cell line or the primary mixed neural cultures.

**Conclusion:**

We demonstrate that the functions of oligodendroglia secreted EVs enriched with HSPB8 have a supportive role, comparable to the native OL-EVs. Further development of engineered oligodendroglia derived EVs could be a novel therapeutic strategy in countering chronic inflammation.

**Video Abstract**

**Supplementary Information:**

The online version contains supplementary material available at 10.1186/s12964-022-00863-x.

## Background

Neuroglial cells, including oligodendroglia and microglia, can secrete extracellular vesicles (EVs) to stimulate cellular debris removal and serve as messengers for neural cell communication [1, 2]. In this regard, EVs may contribute to the development of the central nervous system (CNS), modulate neuroinflammation, and promote regeneration following injury [1]. EVs do not only mediate active processes required for normal CNS function [3], they also contribute in the pathogenesis of many neuroinflammatory disorders by spreading pro-inflammatory signals and altering neuronal functions [4, 5]. Additionally, the exchange of small EVs has also been proposed to be one of the mediators underlying therapeutic activities of mesenchymal stromal cells [6]. Another exciting therapeutic avenue currently being explored is the loading of cell type-specific EVs with repair-promoting molecules targeted to deliver anti-inflammatory and anti-oxidant molecules to the CNS following injury [7, 8].

EVs prepared from in vitro cell cultures have been reported to exhibit therapeutic activities in many preclinical models of immunological and neurodegenerative diseases [9, 10]. For instance, EVs secreted by oligodendroglia (OL) in response to glutamate activation can influence neuronal metabolism and exhibit neuroprotective functions in the acceptor cell [11]. Although OL can trigger protective responses, the unique property of oligodendroglia-derived extracellular vesicles (OL-EVs) to control the inflammatory and therapeutic properties of activated microglia is unclear. Whether OL-EVs can influence activated microglia's phagocytic or autophagic capabilities, via stress proteins clearance of misfolded proteins, remains to be elucidated. Therefore, it is important to investigate autophagy clearance of glial cell communication under proteotoxic stress conditions.

Additionally, in normal cellular signaling and upon neuroinflammation, the process of autophagy responds to oxidative stress [12, 13]. Increased oxidative stress production causes damage to proteins and cellular components, thereby promoting cell death, as observed in various pathologies [14]. Hence, the balance of autophagy-related stress responses and cell death is central to understand redox signaling-related pathogenesis [15]. Dysregulated autophagy, either excessive or impaired, has been linked to neurodegeneration and plays a crucial role in modulating neuroinflammation [16]. We and others have shown that the efficient removal of cytotoxic proteins from neural cells during neurodegeneration involves the activity of small heat shock proteins known to facilitate autophagic degradation of misfolded proteins [17–20].

Heat shock proteins (HSPs) encompass a large family of molecular chaperones that are well-known regulators of proteostasis, protein refolding, and degradation [20, 21]. The small heat shock protein B8 (HSPB8) protects cells against oxidative stress-mediated cell death by supporting autophagic activity [17–19]. HSPB8 and co-chaperone BCL-2-associated anthanogene 3 (BAG3) are involved in chaperone-assisted selective autophagy, promoting the selective degradation of proteins to reduce cell stress [22]. Consequently, secreted HSPB8 can be proposed as a potential therapy to remove toxic proteins and control excessive inflammatory responses. Of note, it is unknown whether EVs loaded with HSPB8, produced in oligodendroglia stably expressing HSPB8 (OL-HSPB8-EVs), retain the beneficial chaperone effects typical of the cytosolic chaperon HSPB8.

We hypothesize that in vitro produced OL-HSPB8-EVs can modulate cell survival pathways by activating autophagy and reducing ROS activities. Therefore, we used a monoculture system to study the oligodendroglia-microglia crosstalk, essential for the prevention and propagation of inflammation in the brain [23]. The human microglial C20 cell line and a mouse primary mixed neural culture were activated with Tumor Necrosis Factor-alpha (TNFα) and Interleukin 1-beta (IL1β) as a model of cellular inflammation [24, 25]. Phorbol-12-Myristate-13-Acetate (PMA) was used as a model of oxidative stress [26, 27]. In this study, the effects of in vitro produced native OL-EVs and modified OL-HSPB8-EVs on neural cells were investigated. We examined how HSPB8 enriched EVs could contribute to a protective effect through autophagy activation, chaperoning support, and protection against oxidative stress.

## Material and methods

### Cell culture material and conditions

The human microglia C20 cell line was donated by Dr. David Alvarez-Carbonell, Case Western Reserve University Cleveland, Ohio. Cells were cultured at 37 °C and 5% CO_2_ in complete medium consisting of Dulbecco’s Modified Eagle’s Medium (DMEM, Sigma-Aldrich, D6429), supplemented with 1 µM dexamethasone (PromoKine, PK-CA577-1042–1), 1% penicillin–streptomycin (Sigma-Aldrich, P4333), and 10% fetal bovine serum (FBS, Biowest, S181B). Hela cells were cultured at 37 °C, and 5% CO_2_ in Minimum Essential Medium (ThermoFisher Scientific, 31095-029) complemented with 1% penicillin–streptomycin (ThermoFisher Scientific, 15140-122), 1% L-glutamate 200 mM (ThermoFisher Scientific, 25030-024), and 10% fetal bovine serum (FBS, Biowest, S181B). Upon EV stimulation, the complete cell culture media supplemented with 10% FBS (Biowest, S181B) was replaced by the EV-depleted media containing 10% EV-depleted serum (System Bioscience, EXO-FBS-250A-1) to avoid bovine derived EV contamination.

### Generation of HSPB8 CRISPR/Cas9 knock-out cell line

HSPB8 knock-out in HeLa cells was performed by CRISPR/Cas9 technology following the protocol described by Ran et al. [28]. In brief, at least two sgRNAs were designed using the online tool from the Broad Institute (https://portals.broadinstitute.org/gpp/public/analysis-tools/sgrna-design), which cuts at least 30 amino acids after the start codon and which had the lowest number of off-target in the coding regions. The sgRNAs were ordered as phosphorylated primers from IDT (Integrated DNA Technologies) and cloned into the pSpCas9(BB)-2A-Puro (PX459) V2.0 (plasmid #62988, Addgene). Constructs were transformed into DG-1 chemo competent bacteria, plated on agar plates, and sequence validated with Sanger sequencing. The plasmid with both the sgRNA of interest and the Cas9 was further used to transiently transfect naïve HeLa cells with Lipofectamine LTX with Plus Reagent (15338500 ThermoFisher Scientific). 24 h after transfection, the medium was refreshed with a medium containing puromycin (1 µg/mL) for 72 h to select the plasmid-containing cells. Surviving cells were serially diluted in a 96-well plate to obtain single colonies. Single clones were expanded, and the complete *HSPB8* knock-out was verified for the presence of a premature stop codon by Sanger sequencing. The absence of the HSPB8 protein was verified by western blotting.

### Generation of human oligodendroglia cell lines stably expressing HSPB8

The human oligodendroglia cell line stably expressing HSPB8 was generated as previously described by Irobi et al. [18]. In brief, constructs encoding the HSPB8 wild-type gene were created using the Gateway recombination technology, sequence validated, and PCR amplified (Invitrogen). The PCR products containing the HSPB8 ORF flanked by the attB recombination sites were then recombined into a pDONR221 vector and validated for the sequence. Next, pDONRs-HSPB8 constructs were transferred by recombination to the pLENTI6/V5-Dest (Invitrogen), generating constructs with the ORFs C-terminally fused to a V5 tag. According to a previously described method, the oligodendroglia cell line was produced by lentiviral transduction [29]. After infection, oligodendroglia was selected in DMEM supplemented with 10% FCS, 1% non-essential amino acids, 2 mm glutamine, and 100 µg/mL penicillin/streptomycin and blasticidin (3 µg/mL) for 3–4 weeks. The whole population of selected cells was pooled for further experiments. After selection, cells were cultured at 37 °C and 5% CO_2_ in complete medium consisting of Dulbecco’s Modified Eagle’s Medium (DMEM, Sigma-Aldrich, D6429), supplemented with 1% penicillin–streptomycin (Sigma-Aldrich, P4333), and 10% fetal bovine serum (FBS, Biowest, S181B).

### Hollow fiber 3D EV production

Wild-type human oligodendroglia and stable HSPB8-overexpressing human oligodendroglia (OL-HSPB8) were used to produce native OL-EVs and modified OL-HSPB8-EVs. According to the manufacturer's instructions, a medium-sized, hollow fiber culture cartridge with a 20 kDa molecular weight cut-off (Fibercell Systems, C2011) was pre-cultured. Cell lines were seeded and expanded in conventional culture flasks, harvested, and 2 × 10^8^ cells were inoculated into the extra-capillary space of the hollow fiber cartridge and allowed to adapt to the bioreactor culture conditions for 11 days. Cells were cultured at 37 °C and 5% CO_2_ in Dulbecco’s Modified Eagle’s Medium (DMEM, Sigma-Aldrich, D6429), supplemented with 1 × penicillin–streptomycin (Sigma-Aldrich, P4333), and 10% fetal bovine serum (FBS, Biowest, S181B). The pump flow rate was set following the manufacturer’s recommendations and media was refreshed after reaching 30% of the initial glucose concentration. Serum-free medium was used in the extra-capillary space, and EV-conditioned medium was collected 3 times a week.

### Animals

C57BL/6 J mice were maintained according to the guidelines of the Belgian Law and the European Council Directive in the animal facility of the Biomedical Research Institute (BIOMED) at Hasselt University. The animals were housed on a 12 h light/dark cycle in standard cages with a controlled room temperature of 22 °C. Food and water were provided ad libitum. Experiments were conducted according to the European Community guiding principles on the care and use of animals and the approval of the Ethical Committee on Animal Research of Hasselt University.

### Isolation and culturing of primary mixed neural culture cells

Primary mixed neural cultures were isolated from wild-type P3 mouse pups. Briefly, the cerebra were acutely isolated from decapitated pups and immediately transferred to ice-cold Dulbecco’s Modified Eagle’s Medium (DMEM, Sigma-Aldrich, D6429), supplemented with 1% penicillin–streptomycin (Sigma-Aldrich, P4333). Next, meninges were removed, and the hemispheres were transferred to fresh ice-cold Dulbecco’s Modified Eagle Medium (DMEM, Sigma-Aldrich, D6429) containing 1% penicillin–streptomycin. The brains were mechanically homogenized, sieved through a 70 μm cell strainer, and centrifuged (300 × g, 10 min, 4 °C). The supernatant was discarded, and the pellet was resuspended in DMEM D6429 supplemented with 10% fetal bovine serum (FBS, Gibco, 26140-079), 10% horse serum (Gibco, 26050-088), and 1% penicillin–streptomycin (Sigma-Aldrich, P4333). Primary mixed neural cultures were obtained by seeding the cell suspension in poly-D-lysine pre-coated (PDL; 20 μg/mL, 1 h coating at 37 °C, Sigma-Aldrich, P6407-5MG) T75 culture flasks for 7 days before changing the medium.

### EV isolation

Isolation and characterization of EVs were performed according to the MISEV 2018 guidelines [30]. In brief, the culture medium was collected and immediately pre-cleared for cells and debris by sequential centrifugation at 300 × g for 10 min, 2000 × g for 10 min, and 10,000 × g for 30 min. To pellet EVs, supernatants were ultra-centrifuged for 3 h at 115,000 × g. All centrifugation steps were performed at 4 °C using an Eppendorf 5804 R centrifuge or a Beckman Optima XPN-80 ultracentrifuge equipped with a Ti70 rotor. EV pellets were washed and collected in phosphate-buffered saline (PBS) and further purified by size-exclusion chromatography (SEC) using chromatography columns (Bio-Rad Laboratories, 732-1010) containing 10 mL Sepharose CL-2B beads (60–200 µm) (GE Healthcare,17-0140-01). The beads were sterilized using 70% ethanol (VWR chemicals, 84857.360) and rinsed with PBS. One mL volume of differential ultra-centrifuged EVs concentrate was loaded on the SEC column, followed by elution with PBS buffer to further purify the EVs. The eluate was collected immediately after EVs loading in 10 sequential fractions of 1 mL flow-through. Based on nanoparticle tracking analysis, collection volumes 4, 5 and 6 contained the highest EV concentration (data not shown). However, Böing et al. reported the presence of protein contaminants co-eluting from collection volume fraction 6 [31]. Therefore, fraction 6 was discarded, and EV-containing fraction 4 and 5 were pooled and up-concentrated using Amicon 10 k filters (Merck Millipore, UFC201024).

### Quantification of EVs

Nanoparticle tracking analysis (NTA) was performed using the NanoSight NS300 system (Malvern Panalytical) equipped with a 532 nm laser to analyze EV concentration and size distribution. EV samples were diluted in PBS (Lonza, 17-516F) over a concentration range between 10 and 100 particles per frame. All settings were manually set at the beginning of the measurements and kept constant during all acquisitions: camera level, 14; camera gain, 1; pump rate, 80; viscosity, 1. A minimum of 3 recordings of 1 min per sample was analyzed by the NTA software 3.0 with default settings.

### Transmission electron microscopy of purified EVs

OL-EV and OL-HSPB8-EV morphology was determined using transmission electron microscopy. Twenty microliter droplets of EVs suspended in PBS were placed on a Parafilm. Next, formvar-nickel TEM slots were placed on top of the EV droplets and allowed to adsorb for 60 min. Slots with adherent EVs were washed and fixed for 10 min with 2% glutaraldehyde. Subsequently, the slots were washed and transferred to droplets of 2% uranyl acetate for 15 min. Finally, the slots were embedded in 0.13% methylcellulose and 0.4% uranyl acetate. After drying, slots were examined with a Tecnai G2 Spirit Bio Twin transmission electron microscope (TEM, FEI/ThermoFisher, Eindhoven, The Netherlands)) at 120 kV.

### Western blotting

EVs and cells were lysed for 30 min in ice-cold RIPA buffer (ThermoFisher, 89900), supplemented with protease (Roche, 05892970001) and phosphatase inhibitor (Roche, 04906845001). The lysate was cleared at 17,000 × g for 15 min. Next, protein concentrations were determined using Pierce BCA protein assay (ThermoFisher Scientific, 23227) according to the manufacturer’s protocol. Proteins were denatured at 95 °C for 5 min with 1 × sample buffer, loaded, and separated on a 12 or 15% SDS-PAGE gel. Subsequently, proteins were transferred for 1 h at 0.4 A on a polyvinylidene difluoride membrane (Immobilon®, IPVH00010) and blocked for 1 h with 5% milk powder in PBS (Lonza, 17-516F) containing 0.1% Tween 20 (VWR, MERC8.22185.0500). Finally, the membranes were incubated with a primary antibody overnight at 4 °C. Horseradish peroxidase-conjugated secondary antibodies (Dako, Denmark) were used for 1 h at room temperature. Proteins were visualized using Pierce ECL Plus Western Blotting Substrate (ThermoFisher Scientific, 32132) for cell lysate or the WesternBright™ Sirius detection kit (Advansta, K-12043-D10) for EV lysate. Band intensities were determined by quantifying the mean pixel gray values using ImageJ software. The following primary antibodies were used: Exosome-anti-CD63 (Invitrogen, 10628D), Exosome-anti-CD9 (Invitrogen, 10626D), anti-Annexin A2 (Cell Signaling Technology, D1162), anti-Flotillin-1 (Cell Signaling Technology, D2V7J), anti-HSP70 (Santa Cruz Biotechnology, sc-32239), anti-Grp94 (Cell Signaling Technology, D6X2Q), anti-HSPB8/HSP22 (Cell Signaling Technology, 3059), anti-LC3B (Sigma-Aldrich, L7543), anti-BAG3 (Protein Tech, 10599-1-AP), anti-SQSTM1/P62 (Cell Signaling Technology, 5114), anti-β-actin (Santa Cruz Biotechnology, sc-47778), anti-ubiquitin (Cell Signaling Technology, 3936). The following secondary antibodies were used: polyclonal Rabbit Anti-Mouse Immunoglobulin/HRP (Dako, P0260) and polyclonal Goat Anti-Rabbit Immunoglobulin/HRP (Dako, P0448).

### Real-time quantitative PCR

2 × 10^5^ C20 cells were seeded in 6-well plates containing complete medium. After 24 h, the complete medium was replaced with EV-depleted medium, and cells were stimulated with 5 × 10^9^ OL-EVs or OL-HSPB8-EVs for 24 h, followed by 100 ng/mL TNFα (Immunotools, 11343015) and 100 ng/mL IL1β (Immunotools, 11340015) activation for 24 h. As positive controls, PMA (100 ng/mL, 15 min), rapamycin (10 µM, 24 h), and staurosporine (2 µM, 3 h) were used. Subsequently, cells were harvested, and total RNA was prepared using the RNeasy Plus mini kit (Qiagen, 74106) according to the manufacturer’s instructions. RNA quality and quantity were determined using a NanoDrop spectrophotometer (Isogen Life Science, IJsselstein, The Netherlands). Further, RNA was reverse transcribed using the qScript™ cDNA SuperMix (Quanta Biosciences, 95048), and quantitative PCR was performed on a StepOnePlus™ RT-PCR System (Applied Biosystems, Halle Belgium). Fast SYBR Green (Applied Biosystems, 4385612), 10 µM forward and reverse primer mixture (Integrated DNA Technologies, Leuven, Belgium), and 10 ng RNA were used per reaction. Relative quantification of gene expression was accomplished using the ΔΔCt method. Expression levels were normalized using the most stable housekeeping genes; GAPDH and RPL13a as determined with geNorm. Primer sequences can be found in Additional file 1.

### EV uptake by flow cytometry

C20 cells were seeded in 24-well plates at a density of 1 × 10^5^ cells per well. Primary mixed neural culture cells were seeded in a 96-well plate at a density of 1.5 × 10^5^ cells per well. After 24 h, cells were washed with PBS (Lonza, 17-516F) and activated with TNFα (100 ng/mL, Immunotools, 11343015) and IL1β (100 ng/mL, Immunotools, 1134O015) for 24 h to mimic inflammation. Next, cells were stimulated with 5 × 10^9^ DiI-labelled EVs/mL in EV-depleted medium for 3 h at 37 °C to allow uptake. Equal volumes of OL-EVs and OL-HSPB8-EVs were labeled with 555 nm Vybrant DiI Cell-Labeling Solution (Life Technologies, 15704352) for 30 min at 37 °C. Unincorporated dye was removed by SEC. NTA was performed to determine EV concentrations. After incubation, cells were harvested using trypsin EDTA (Sigma-Aldrich, T3924) and centrifuged for 10 min at 300 × g. The supernatant was discarded, and cell pellets were resuspended in 500 µL PBS containing 4% FBS (Biowest, S181B). Single-cell mean fluorescent intensities were quantified at a wavelength of 555 nm by flow cytometry on a BD-LSRFortessa flow cytometer (BD Biosciences) using FACSDive software (BD Biosciences).

### Chaperone activity assay

Naïve and HSPB8 KO HeLa cells were seeded in 6-well plates containing a complete medium. At 70% confluence, the complete medium was replaced by an EV-depleted medium, and cells were stimulated for 48 h either with 2 × 10^9^, 5 × 10^9^, 10 × 10^9^ OL-EVs/mL, or OL-HSPB8-EVs/mL. After stimulation, cells were heat-shocked for 90 min at 42 °C, collected in ice-cold PBS, and pelleted by centrifugation. Subsequently, the pellets were resuspended in NP-40 lysis buffer (150 mM NaCl, 20 mM TrisBase, NP-40 0.05%, 1.5 mM MgCl_2_, Glycerol 3%, pH 7.4) supplemented with 1 M DTT and 1% Halt™ protease inhibitor (Thermo scientific, 78429), passed through a 23G 0.6 mm syringe 10 × and immediately centrifuged (16,100 × g, 15 min, 4 °C). The supernatant (soluble fraction) was collected, the pellet (insoluble fraction) was washed twice in ice-cold PBS, centrifuged (16,100 × g, 4 min, 4 °C), resuspended in equal volume used to lyse the cells of NP-40 buffer without DTT, and sonicated 10x (50% amplitude, 0.5 cycles) using a UP50H Ultrasonic Processor (Hielscher ultrasonics GmbH, Teltow, Germany). Next, from the soluble fraction, protein concentrations were determined using Pierce BCA protein assay (ThermoFisher Scientific, 23227) according to the manufacturer’s protocol. Proteins were denatured at 95 °C for 5 min with 1 × sample buffer, loaded, and separated on a 4–12% SDS-PAGE gel (NP0322BOX, Life Technologies). Running, transferring, blocking, and antibody incubation were performed as described previously.

### Immunocytochemistry and confocal microscopy

Immunostainings were performed according to standard protocols. 5 × 10^4^ C20 cells were seeded on a glass cover slide placed in a 24-well plate and submerged in complete medium. After 24 h, the complete medium was changed to EV-depleted medium, and cells were stimulated with either 5 × 10^9^ OL-EVs/mL or 5 × 10^9^ OL-HSPB8-EVs/mL. After 24 h, cells were activated with TNFα (100 ng/mL, Immunotools, 11343015) and IL1β (100 ng/mL, Immunotools, 11340015) to mimic inflammation. Autophagy was induced using 10 µM rapamycin (Enzo, ENZ-51031) for 24 h, and lysosomal degradation was inhibited using 10 µM chloroquine (Enzo, 51005-CLQ) for 18 h. Cells were fixed in ice-cold 100% methanol (VWR, 30847) for 15 min and permeabilized using 0.1% Triton X-100 (VWR, 437002A) in PBS (Lonza, 17-516F) for 15 min at 4 °C. Blocking was performed with 5% BSA (Life Sciences, A1324) diluted in PBS for 1 h. The primary LC3B antibody was incubated overnight at 4 °C, and the secondary antibody was incubated for 1 h, diluted in 5% BSA in PBS supplemented with 0.1% Tween 20 (VWR, MERC8.22185.0500) at room temperature. Nuclear staining was performed using DAPI (Thermo Fisher Scientific, 62248). Next, cells were mounted with a fluorescent mounting medium (Thermo Scientific, 9990402) and images were taken on a Zeiss LSM880 Airyscan laser scanning confocal microscope. All images used for quantification were acquired using identical fluorescence excitation and detection settings that avoided channel crosstalk. Three replicate wells were used per condition. An image analysis pipeline was designed in CellProfiler to automatically quantify the number of punctate structures (LC3B, mean fluorescence intensity) per individual cell. In short, nuclei were segmented based on the DAPI channel (Adaptive thresholding Otsu method) and used as seeds to segment the cells (Propagation method with global Otsu thresholding). Punctate structures were segmented in the appropriate channel using the global MoG thresholding channel. Standard CellProfiler modules were used to relate the different masks, measure shape, and intensity parameters, and create output tables.

### CYTO ID autophagy assay

Autophagy induction was determined by using the CYTO-ID Autophagy Detection Kit (Enzo, ENZ-51031) according to the manufacturer’s protocol. Briefly, 1 × 10^5^ C20 cells or 2 × 10^5^ primary mixed neural culture cells were seeded in 24-well or 96-well plates containing complete medium, respectively. The next day, the complete medium was changed to EV-depleted medium, and cells were stimulated for 48 h with either 5 × 10^9^ OL-EVs/mL or 5 × 10^9^ OL-HSPB8-EVs/mL. At 24 h after EV stimulation, cells were activated for 24 h by a mixture of 100 ng/mL TNFα (Immunotools, 11343015) and 100 ng/mL IL1β (Immunotools, 11340015) to mimic inflammation or cells were stimulated with 10 µM rapamycin (Enzo, ENZ-51031) for 24 h to induce autophagy. Lysosomal degradation was blocked using 10 µM chloroquine (Enzo, 51005-CLQ) for 18 h. After incubation, cells were trypsinized, centrifuged for 10 min at 300 × g, PBS-washed, again centrifuged, and resuspended in 0.5 mL assay buffer. Subsequently, cells were stained with the CYTO-ID green detection dye for 30 min at 37 °C in the dark. After staining, the cells were pelleted at 300 × g for 10 min and resuspended in 0.5 mL assay buffer. Single-cell mean fluorescent intensities were quantified at a wavelength of 488 nm by flow cytometry on a BD-LSRFortessa flow cytometer (BD Biosciences) using FACSDive software (BD Biosciences).

### Transmission electron microscopy assessment of autophagy

Human microglia C20 cells (5 × 10^4^) were grown in 8-well chambered Permanox slides (Nunc, Lab-Tek, C7182-1PAK) and stimulated with 5 × 10^9^ OL-EVs/mL or OL-HSPB8-EVs/mL in 400 µL EV-depleted medium. As a control, cells were stimulated with 10 µM rapamycin (Enzo, ENZ-51031) for 24 h to induce autophagy. Lysosomal degradation was blocked using 10 µM chloroquine (Enzo, 51005-CLQ) for 18 h. After stimulations, C20 cells were fixed in 0.1 M sodium cacodylate-buffered, pH 7.4, 2.5% glutaraldehyde solution for 2 h at 4 °C and washed in 0.1 M sodium cacodylate, pH 7.4 (Sigma-Aldrich, 6131–99-3) containing 7.5% saccharose (Sigma-Aldrich, 57-50-1). Next, cells were incubated for 1 h in 1% OsO_4_ solution (Sigma-Aldrich, 20816-12-0). After dehydration in an ethanol gradient, cells were embedded in EM-bed812 (Electron Microscopy Sciences, EMS14120). Ultrathin sections were stained with lead citrate and samples were examined in a Tecnai G2 Spirit Bio Twin Microscope (FEI, Eindhoven, The Netherlands) at 120 kV. Quantification of autophagic vesicles was achieved by manually counting vesicles. Images were blinded and randomly taken at a magnification of 26,000 × in the cytoplasm of the cells. Autophagic vesicles were identified based on ultrastructural characteristics as described by Ylä-Anttila et al. [32].

### ROS-DCFH assay

ROS activity was measured by seeding 1 × 10^5^ C20 cells or 2 × 10^5^ primary mixed neural culture cells in 24-well or 96-well plates containing complete medium, respectively. The next day, cells were stimulated with 5 × 10^9^ OL-EVs/mL or 5 × 10^9^ OL-HSPB8-EVs/mL in an EV-depleted medium. Subsequently, cells were left non-activated, activated with 100 ng/mL TNFα (Immunotools, 11343015) and 100 ng/mL IL1β (Immunotools, 11340015) for 24 h or 100 ng/mL PMA (Merck, P1585) for 15 min. After stimulations, cells were harvested using trypsin, centrifuged, and stained with 10 µM 2′,7′-dichlorofluorescein diacetate (DCFH-DA, Enzo, ALX-610–022-M050) solution for 30 min at 37 °C in the dark. Subsequently, cells were pelleted, washed, and resuspended in PBS containing 4% FBS (Biowest, S181B). Single-cell mean fluorescent intensities were quantified at a wavelength of 488 nm by flow cytometry on a BD-LSRFortessa flow cytometer (BD Biosciences) using FACSDive software (BD Biosciences).

### JC-1 mitochondrial membrane potential assay

The JC-1 Mitochondrial Membrane Potential Assay Kit (BD Biosciences, 551302) was used according to the manufacturer's protocol. In brief, 1 × 10^5^ C20 cells or 2 × 10^5^ primary mixed neural culture cells were seeded in 24-well or 96-well plates containing complete medium, respectively. After 24 h, the complete medium was changed to EV-depleted medium, and cells were stimulated with 5 × 10^9^ OL-EVs/mL or OL-HSPB8-EVs/mL, followed by TNFα (100 ng/mL, Immunotools, 11343015) and IL1β (100 ng/mL, Immunotools, 11340015) activation for 24 h or PMA (100 ng/mL, Merck, P1585) stimulation for 15 min. Subsequently, cells were harvested, centrifuged, PBS-washed, again centrifuged, and suspended in 0.5 mL assay buffer. Next, cells were stained with the JC-1 green detection dye for 30 min at 37 °C in the dark. After centrifugation, the supernatant was discarded, and the pellet was resuspended in 0.5 mL assay buffer. Single-cell mean fluorescent intensities were quantified at a wavelength of 488 nm and 594 nm by flow cytometry on a BD-LSRFortessa flow cytometer (BD Biosciences) using FACSDive software (BD Biosciences).

### Annexin V phosphatidylserine (PS) apoptosis assay

Phosphatidylserine (PS) exposure was explored using the PS-binding protein Annexin V conjugated to a fluorescent FITC label to determine early apoptosis. 2 × 10^5^ primary mixed neural culture cells or 1 × 10^5^ C20 cells were seeded in 96-well or 24-well plates containing complete medium, respectively. The next day, the complete medium was switched to EV-depleted medium, and cells were stimulated with 5 × 10^9^ OL-EVs/mL or OL-HSPB8-EVs/mL for 24 h, followed by TNFα/IL1β (100 ng/mL, Immunotools 11343015, 11340015) activation for 24 h. Apoptosis was induced using 2 µM staurosporine (Enzo, ALX-380-014-M001) for 3 h. Cells were collected, centrifuged, and resuspended in 100 µL of assay buffer (Invitrogen, PNN1001). Per reaction, 5 µL Annexin V-FITC (Invitrogen, 11-8005-74) and 2.5 µL 7AAD dye (BD Pharmingen, 51-68981E) were incubated for 15 min in the dark at room temperature. Next, cells were centrifuged, washed, and resuspended in 1 × assay buffer. Single-cell fluorescent intensities were quantified by flow cytometry on a BD-LSRFortessa flow cytometer (BD Biosciences) using FACSDive software (BD Biosciences).

### Statistical analysis

Statistical analyses were performed using Student’s two-tailed t-test or one-way ANOVA unless noted otherwise. Data were analyzed using the GraphPad Prism software and presented as the mean ± SEM. Values of *P* < 0.05 were considered statistically significant.

## Results

### Characterization of native and HSPB8-loaded oligodendroglia-derived small extracellular vesicles

**Fig. 1:**
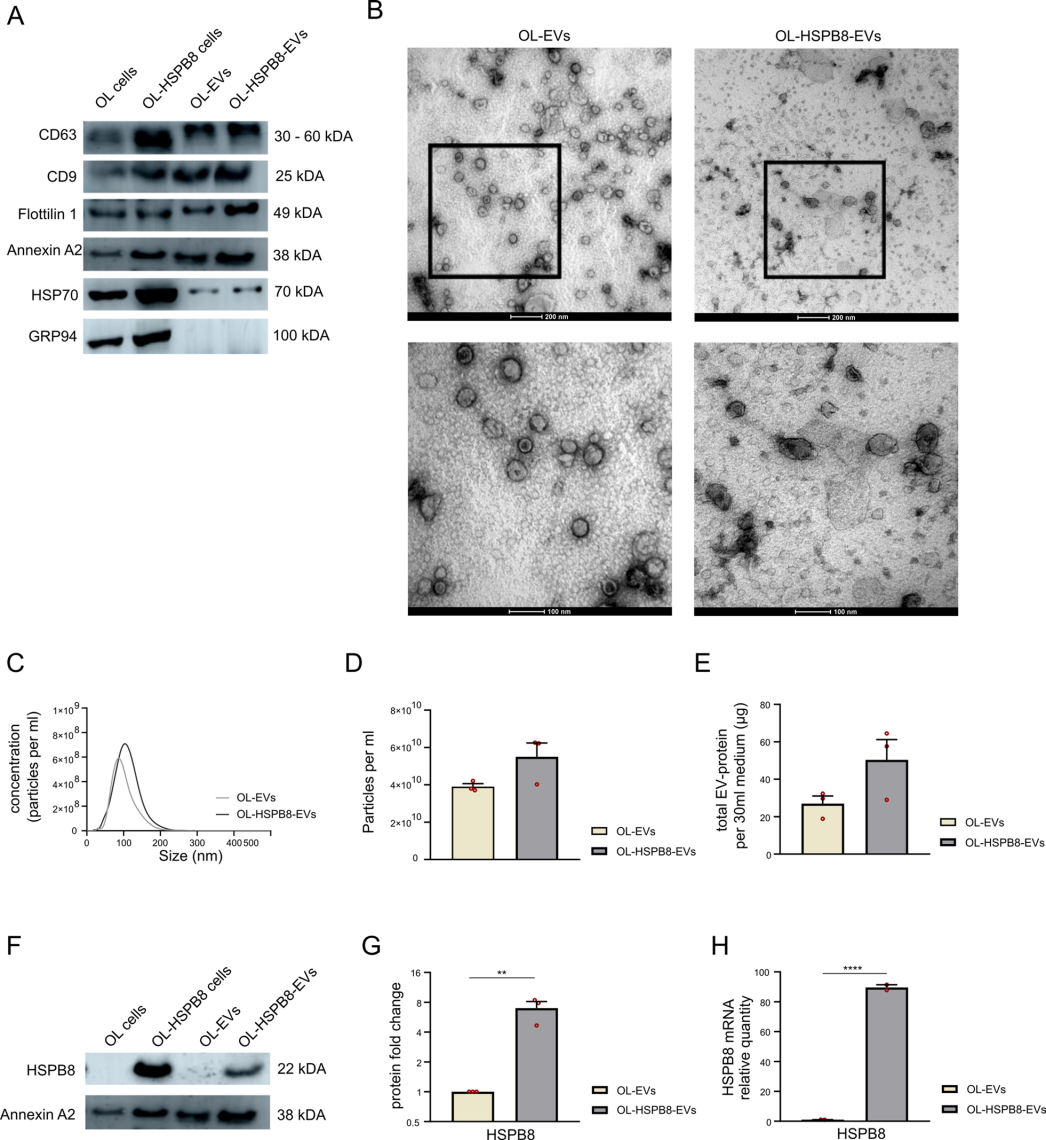
Characterization of human oligodendroglia-derived extracellular vesicles. **A** Western blotting analysis of small extracellular vesicle protein markers CD63, CD9, Flotillin-1, Annexin A2, HSP70, and the negative control marker GRP94. **B** Representative overview (scale bar 200 nm) and cropped (scale bar 100 nm) image showing the morphology, as revealed by transmission electron microscopy, of EVs isolated from wild-type native human oligodendroglia (OL-EVs) and stably expressing HSPB8 oligodendroglia (OL-HSPB8-EV). **C**–**E** Particle size profile distribution, particles per mL, and total EV protein measured by Nanoparticle Tracking Analysis (NTA) and BCA of OL-EVs and OL-HSPB8-EVs, pelleted from 30 mL of conditioned medium. **F**–**H** Western blotting and qPCR analysis of HSPB8 present in OL-EVs and OL-HSPB8-EVs. Statistical analysis was performed using GraphPad. Student’s t-test was used to determine significance (**P < 0.01, ****P < 0.0001). Data are presented as mean ± SEM

Human OL cell lines were either genetically modified to stably express HSPB8 (OL-HSPB8-EVs) or remained unmodified (native OL-EVs). The isolated OL-EVs subsets were characterized according to the MISEV 2018 guidelines [30]. Immunoblotting analysis revealed the presence of the EV markers CD63, CD9, Flotillin-1, Annexin-A2, and HSP70 in OL-EVs and OL-HSPB8-EVs. To confirm EV purity, the EV-negative marker GRP94 was analyzed. Our data indicate the absence of the endoplasmic marker GRP94 in both OL-EVs and OL-HSPB8-EVs protein lysate (Fig. 1A). Transmission electron microscopy (TEM) images revealed that both OL-EVs and OL-HSPB8-EVs had a similar cup-shaped or spherical morphology (Fig. 1B). Additionally, nanoparticle tracking analysis (NTA) showed that the distribution curves of the OL-EV and OL-HSPB8-EV particle size were similar with a mean particle size of 100.9 nm and 111.2 nm, respectively (Fig. 1C). As quantified by NTA and BCA, results demonstrate a non-significant difference in particles per mL and total EV protein concentration between OL-HSPB8-EVs and OL-EVs (Fig. 1D, E). To determine the enrichment of HSPB8 in OL-HSPB8-EVs, Western blotting and quantitative real-time PCR (qPCR) were performed on the lysate of both EV subsets. HSPB8 protein and mRNA quantification showed significantly increased levels in OL-HSPB8-EVs compared to OL-EVs (Fig. 1F–H). Together, these analyses confirmed the successful isolation of both the native OL-EVs and the HSPB8-enriched EVs from human OL cell culture media.

### OL-HSPB8-EVs internalized by microglia and primary mixed neural culture cells increases HSPB8 expression and modulates chaperone activity

**Fig. 2:**
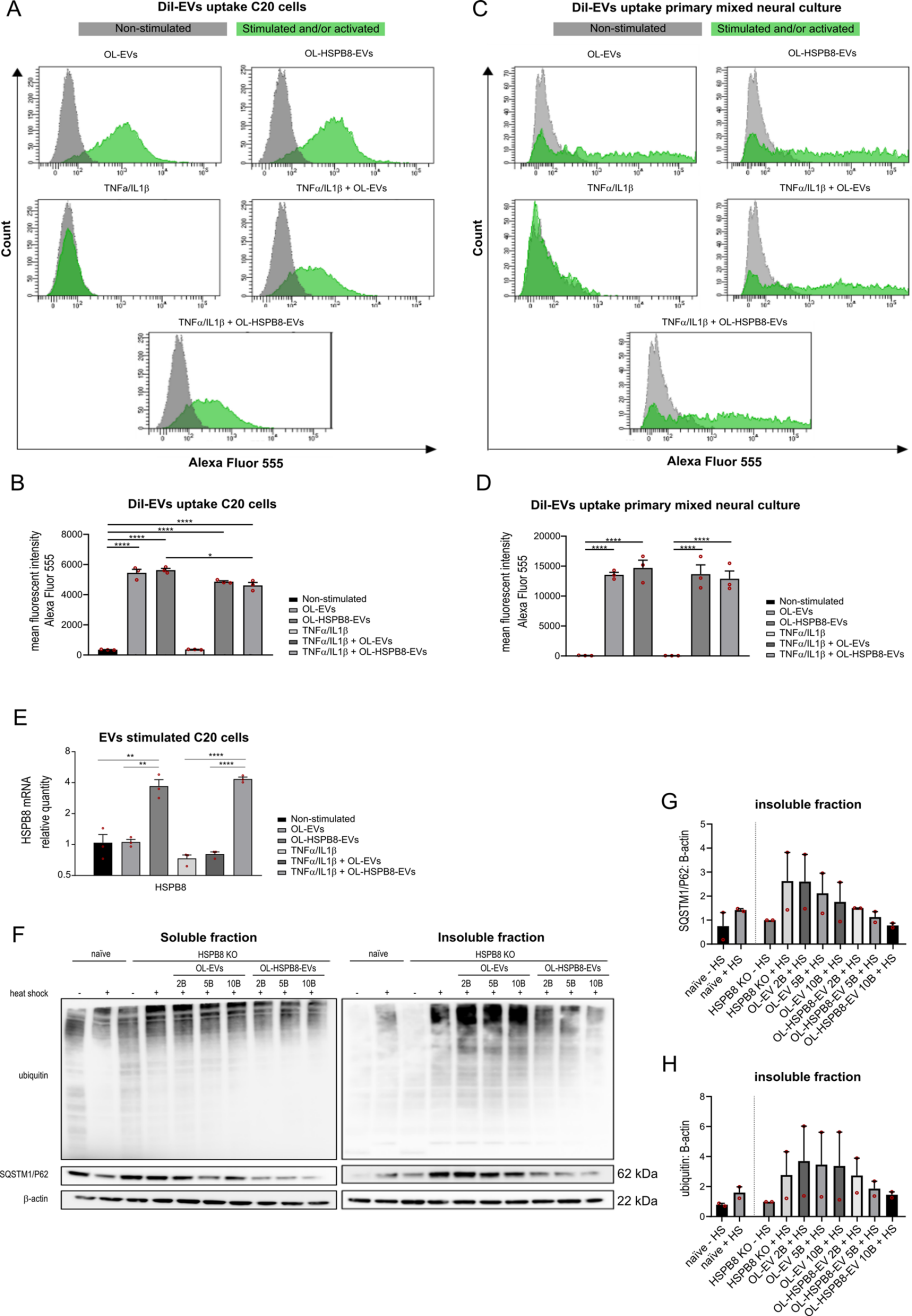
OL-EVs and OL-HSPB8-EVs uptake and chaperone activity. **A**–**D** Quantification of cellular uptake of DiI-labelled OL-EVs and OL-HSPB8-EVs by flow cytometry in human microglia C20 and primary mixed neural cultures. The data presents the mean fluorescent intensity and are shown as mean ± SEM (n = 3 biological replicates per group. Green peaks show live cells stimulated with DiI-labelled EVs and/or activated with TNFα/IL1β while grey peaks show the non-stimulated, non-activated live cells **E** Relative qPCR expression of HSPB8 in human microglia C20 cell line stimulated with OL-EVs or OL-HSPB8-EVs either non-activated or TNFα/IL1β activated. Data were analyzed using stable reference genes, and the fold changes were plotted as the mean ± SEM and normalized to the non-stimulated control cells (n = 3 biological replicates per group). **F**–**H** Western blotting analysis of SQSTM1/P62 and ubiquitinated proteins in HeLa HSPB8-KO cells in the soluble (non-aggregated) and insoluble fraction (aggregated). The cells were either OL-EVs, OL-HSPB8-EVs stimulated or non-stimulated and exposed to a 90 min heat shock at 42 °C. Protein levels were calculated from two independent experiments and normalized to β-actin. Band intensities were determined by quantifying the mean pixel gray values using the ImageJ software. Data are presented as mean ± SEM. One-way ANOVA was used in GraphPad to determine statistical significance (*P < 0.05, **P < 0.01, ***P < 0.001, ****P < 0.0001)

In order to characterize the biological activities of OL-EVs in human and mouse cells, the internalization of OL-EVs and OL-HSPB8-EVs into human microglial C20 cells and primary mixed neural culture cells was investigated. EVs derived from an OL donor cell can be incorporated into a recipient cell by a paracrine mechanism of action [33]. To examine the uptake of OL-EVs and OL-HSPB8-EVs, we investigated whether fluorescent DiI-labelled EVs could be incorporated into the different cell types under normal or inflammatory conditions. Cellular uptake of fluorescently labeled EVs was evaluated by flow cytometry. Results show that both subsets of DiI-labelled EVs (OL-EVs and OL-HSPB8-EVs) were incorporated into microglial C20 cells (Fig. 2A) and primary mixed neural culture cells (Fig. 2C). A significant increase in DiI fluorescence was detected in cells incubated with the labelled OL-EVs or OL-HSPB8-EVs compared to cells without DiI labelled EVs. This result supports the interaction between labelled EVs and the recipient cells (Fig. 2B, D). Similar internalization of EVs was observed in both non-activated and TNFα/IL1β activated cell conditions (Fig. 2B, D). Of note, the same EV concentration of 5 × 10^9^ per mL was used in all experiments. Overall, no difference in cellular uptake was observed between OL-EVs and OL-HSPB8-EVs in either the microglial C20 cells or the primary mixed neural culture cells (Fig. 2B, D).

To investigate whether the internalized OL-EVs could induce changes in the mRNA expression of the HSPB8 gene, a qPCR experiment on microglial C20 cells following EV stimulation was performed. We observed a significant increase in HSPB8 mRNA when cells were stimulated with OL-HSPB8-EVs compared to OL-EVs and control cells (Fig. 2E). Additionally, a similar increase in HSPB8 protein level was observed after OL-HSPB8-EVs stimulation in non-activated conditions compared to OL-EVs and control cells (Additional file 2A-B).

Furthermore, we examined the effect of the EV subsets on the chaperone activity following temperature-induced aggregation. HeLa HSPB8 knock-out (KO) cells were first stimulated with either OL-EVs, OL-HSPB8-EVs, or non-stimulated and then heat-shocked for 90 min at 42 °C. As a marker of protein aggregation, ubiquitinated proteins and Sequestosome-1 (SQSTM1/p62) were detected in the cells' insoluble fraction. Immunoblot analysis of the chaperone assay revealed that the soluble fraction showed no overt changes in ubiquitinated proteins and SQSTM1/P62 levels upon heat shock. In contrast, an increase in aggregated SQSTM1/P62 and ubiquitinated proteins was observed in the insoluble fraction of naïve and HeLa HSPB8 KO cells upon heat shock (Fig. 2F–H). Interestingly, HSPB8 depletion causes a stronger enrichment in aggregated protein in the insoluble fraction compared to the naïve Hela cells, suggesting the importance of HSPB8 in maintaining the protein solubility in stress conditions. Furthermore, after a heat shock, the OL-EVs or OL-HSPB8-EVs stimulation induces a decrease in aggregated SQSTM1/P62 and ubiquitinated proteins compared to the control HeLa HSPB8 KO cells (Fig. 2F–H). OL-EVs or OL-HSPB8-EVs stimulation yielded a non-significant reduction in aggregated proteins. However, we observed an EV dose-dependent reduction in SQSTM1/P62 and ubiquitinated protein aggregation upon heat shock in the insoluble fraction of OL-EVs or OL-HSPB8-EVs stimulated cells. This result suggests that both subsets of oligodendroglia-EVs can reduce the accumulation of aggregated proteins under stress, with OL-HSPB8-EVs exhibiting a superior capacity to counteract the protein stress.

Taken together, these results show that native and OL-HSPB8-EVs can be internalized into microglial cell lines and primary mixed neural culture cells. Additionally, the OL-HSPB8-EVs’ molecular cargo content can increase HSPB8 gene expression and modulate chaperone-mediated clearance of misfolded proteins following heat shock stress by reducing the aggregation of cellular proteins.

### OL-EVs and OL-HSPB8-EVs cargo conveys LC3B-II and BAG3 proteins and activates autophagy in the microglial cell line and primary mixed neural culture cells

**Fig. 3:**
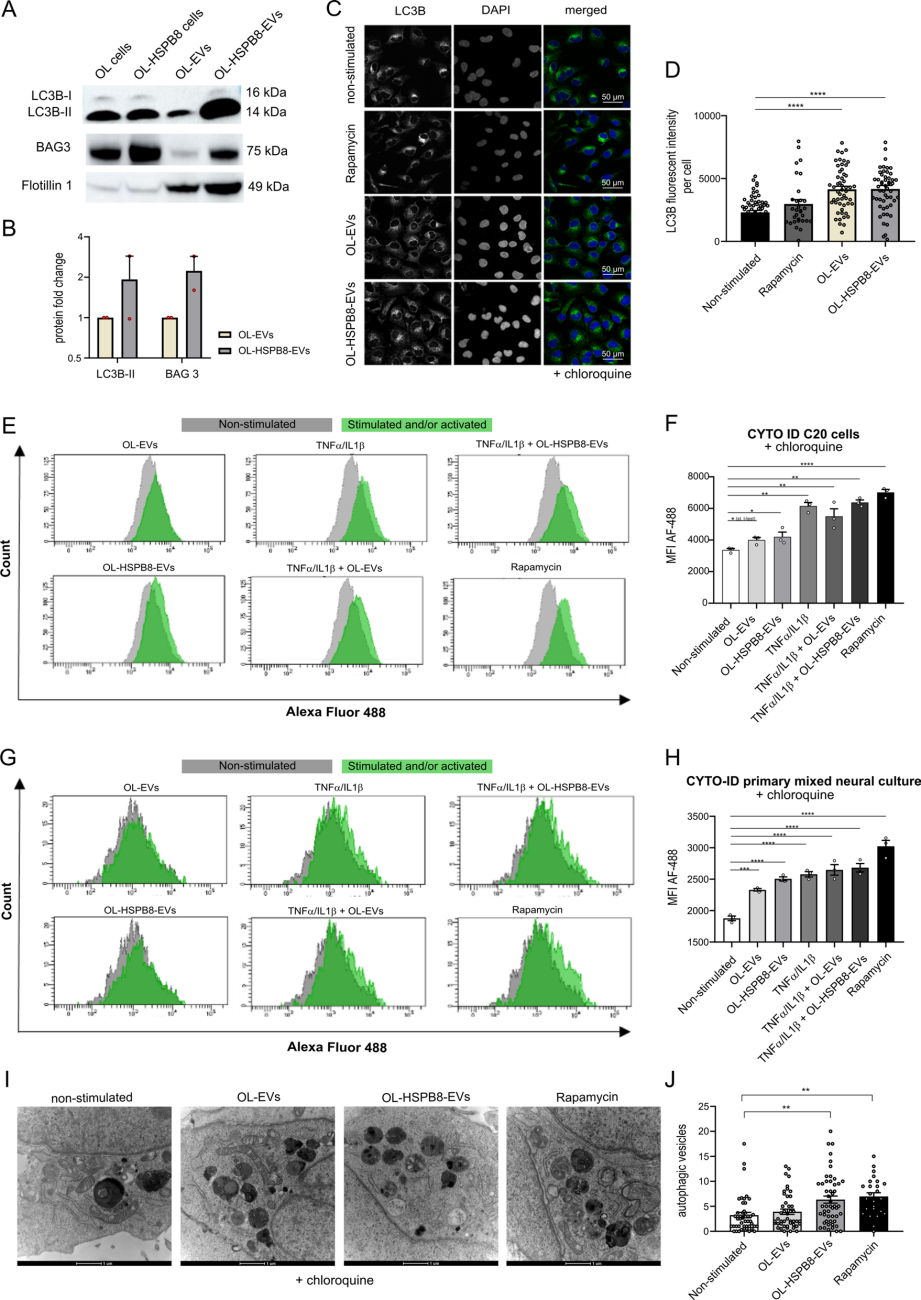
OL-EVs and OL-HSPB8-EVs stimulation activate autophagy in human microglia and primary mixed neural cells. **A**, **B** Western blotting analysis of LC3B-II and BAG3 in OL-EVs and OL-HSPB8-EVs. Protein levels were calculated from two independent experiments and normalized to the EVs marker flotillin-1. Band intensities were determined by quantifying the mean pixel gray values using the ImageJ software. Data are presented as mean ± SEM. **C**, **D** Immunofluorescence staining of human microglial C20 cells stimulated with OL-EVs or OL-HSPB8-EVs. Cells were treated with chloroquine to inhibit lysosomal degradation. Rapamycin was used as a positive control. Representative image showing LC3B (green), the DAPI nucleus stain (blue), and merged images. CellProfiler was used to quantify the LC3B punctate structures (LC3B, mean fluorescence intensity) (n = 4 biological replicates, 15 + cells per experiment). Data are expressed as mean ± SEM, ****P < 0.0001. **E**–**H** Flow cytometry monitoring CYTO-ID autophagic flux in chloroquine treated human microglia C20 cell line and a primary mixed neural culture using the CYTO-ID green detection dye, selectively labeling autophagic vacuoles. Data are presented as mean fluorescent intensities ± SEM (n = 3 biological replicates per group), Green peaks represent TNFα/IL1β activated and/or EV stimulated cells, while the grey peaks show cells of non-activated and non-stimulated control cells. GraphPad was used to determine significance (*P < 0.05, **P < 0.01, ***P < 0.001, ****P < 0.0001). **I**, **J** Ultrastructural analysis of autophagic vesicles by transmission electron microscopy of C20 cells stimulated with OL-EVs or OL-HSPB8-EVs. Lysosomal degradation was blocked using chloroquine. Rapamycin was used as a positive control. The total number of autophagic vesicles per field of view were counted and expressed per cytoplasmic area covered (scale bar = 1 µm). Data are shown as mean ± SEM. GraphPad was used for statistical analysis by using one-way ANOVA (**P < 0.01)

A recent report describes a secretory autophagy pathway in which microtubule-associated protein 1 light chain (MAP1LC3/LC3) mediates the loading of protein and RNA cargoes into EVs [34]. Since our internalized EVs reduced heat shock-induced protein aggregation and contributed to the chaperone activity, we explored whether autophagy is activated due to the EVs stimulation. To examine the presence of the LC3B protein within our purified OL-EVs and OL-HSPB8-EVs, an immunoblot analysis on EV total protein lysates was performed. Results show that the LC3B-II protein was loaded into both the OL-EVs and the OL-HSPB8-EVs. Semi-quantification of LC3B-II Western blots showed an increased protein level in OL-HSPB8-EVs compared to OL-EVs (Fig. 3A, B). Additionally, HSPB8 is part of the chaperone-assisted-selective-autophagy (CASA) complex with the co-chaperone BAG3, which stimulates autophagy in cells [35]. Indeed, immunoblot analysis also revealed BAG3 in both OL-EVs and OL-HSPB8-EVs (Fig. 3A, B), with increased BAG3 levels in OL-HSPB8-EVs compared to OL-EVs. These observed results clearly suggest that OL-EVs and OL-HSPB8-EVs convey LC3B-II and BAG3 autophagy markers.

To investigate whether the internalized EVs could modify autophagy activities in non-activated and TNFα/IL1β-activated cells, we assessed the effect of OL-EVs and OL-HSPB8-EVs incorporated in the microglial C20 cell line and in primary mixed neural culture cells. Microglial C20 cells were stimulated with or without OL-EVs and OL-HSPB8-EVs. Afterward, immunofluorescence analysis was performed for the autophagy marker LC3B, which is usually converted from the LC3B-I form to the lipidated LC3B-II isoform, correlating with the total number of autophagosomes [36]. As a control group, cells were left non-stimulated (negative control) or treated with rapamycin, an autophagy inducer (positive control). The lysosomal blocker chloroquine was used to inhibit lysosomal degradation. Compared to the non-stimulated control group, both OL-EVs and OL-HSPB8-EVs exhibited increased levels of LC3B- punctate structures (LC3B, mean fluorescence intensity) in human microglial C20 cells (Fig. 3C, D). In line with this observation, C20 cells stimulated with OL-EVs and OL-HSPB8-EVs, either activated or non-activated with TNFα/IL1β, displayed a slightly higher LC3B-II protein level and a significant increase in HSPB8 levels on Western blots compared to non-stimulated control cells (Additional file 2). In the same experimental setup, a non-significant increase in BAG3 and SQSTM1/p62 protein levels were observed upon OL-EVs, and OL-HSPB8-EVs stimulation to microglial C20 cells, either in TNFα/IL1β activated or non-activated conditions (Additional file 2).

We further evaluated the effect of EV subsets on the autophagic flux using the flow cytometry quantitative Cyto-ID® Autophagy Detection Kit in microglia C20 cells and in primary mixed neural culture cells stimulated with chloroquine. As expected, a marked increase was observed for both the OL-EVs and OL-HSPB8-EVs stimulated microglial C20 cells compared to non-stimulated control cells (Fig. 3E, F). The same experiment was validated in a primary mixed neural culture, and results show that both EV subsets significantly activate autophagy in primary cells (Fig. 3G, H). However, in TNFα/IL1β activated cells, stimulation with EVs did not show a further increase in autophagic flux when compared to activated cells without EV stimulation. These results indicate that the OL-EVs and OL-HSPB8-EVs cargo contains the BAG3/LC3B protein complex, which can induce LC3B-positive autophagosome formation in cells to maintain cellular homeostasis.

Considering that electron microscopy remains one of the most accurate methods to detect and quantify autophagy, we performed ultrastructural analysis of autophagosomes with transmission electron microscopy to examine the effect of OL-EVs and OL-HSPB8-EVs on autophagy activation in human microglia C20 cells. Chloroquine was used to inhibit lysosomal degradation by preventing the maturation of autophagic vacuoles and inhibiting the fusion of autophagosomes and lysosomes. The autophagy inducer rapamycin was used as a positive control (Fig. 3I, J). We observed an abundant amount of small autophagic vesicles (diameters ranging from 0.2 µm – 1.0 µm) at various stages of degradation, including autophagosomes and autolysosomes (Fig. 3I, J). Quantitative analysis shows that the number of autophagic vesicles per 50 μm^2^ cytoplasm area was significantly higher in the OL-HSPB8-EV-stimulated cells than in the non-stimulated control cells (Fig. 3J). Quantification did not reveal a significant difference in autophagic vesicle number between the OL-EVs and OL-HSPB8-EVs- treated groups (Fig. 3J). Nevertheless, our results suggest that OL-HSPB8-EVs induce autophagy in microglia in vitro.

### OL-HSPB8-EVs reduce PMA-induced oxidative stress in activated microglial cell lines and primary cells

**Fig. 4:**
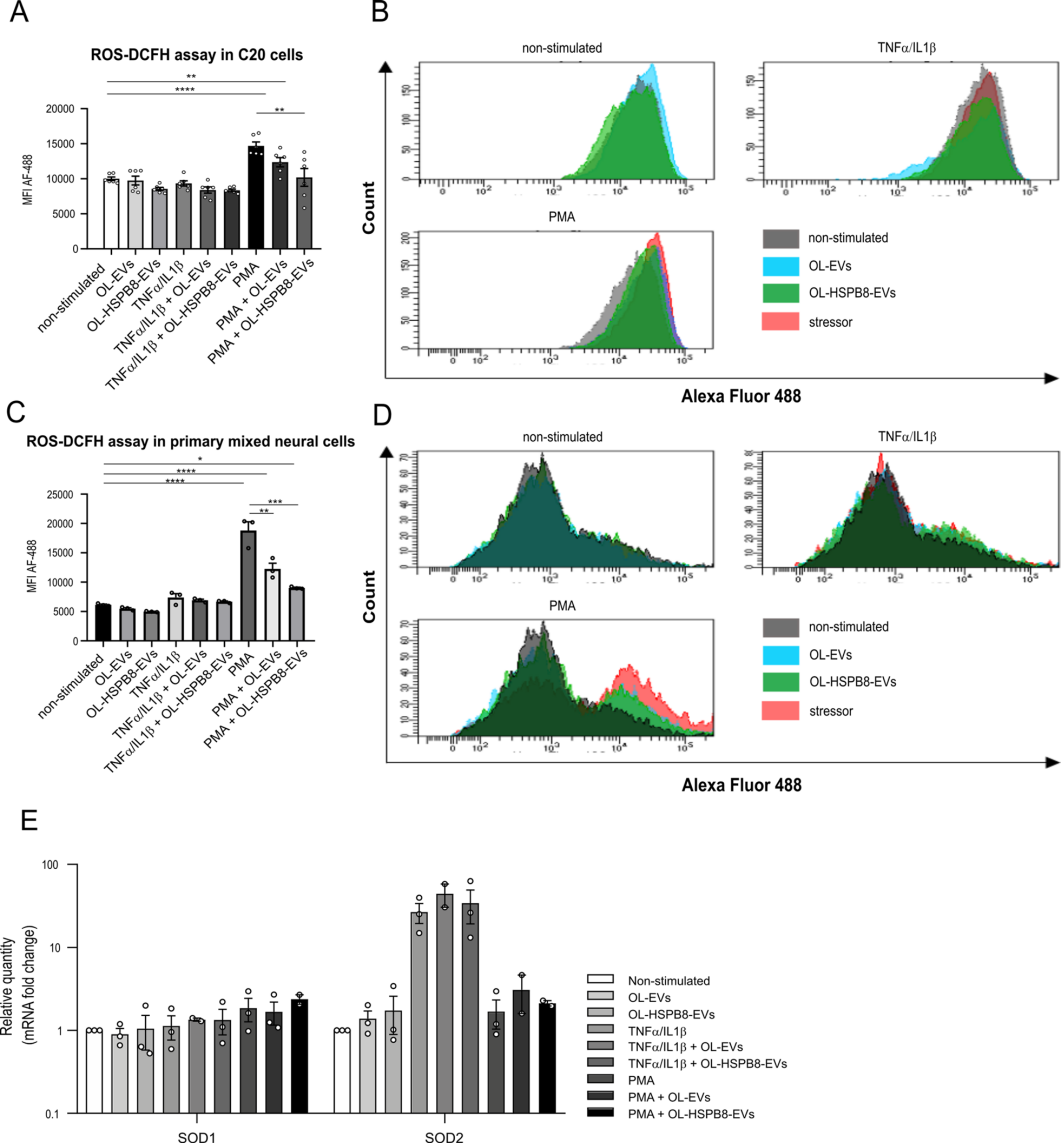
OL-HSPB8-EVs reduce reactive oxygen species upon PMA stimulation. **A**–**D** Flow cytometry monitoring of ROS activity assay in human microglia C20 cell line (**A**, **B**) and a primary mixed neural culture (**C**, **D**) using the DCFH-DA detection dye. Data are presented as mean fluorescent intensities ± SEM (n = 3 biological replicates per group). Graphpad was used to determine significance by using a one-way ANOVA statistical analysis (*P < 0.05, **P < 0.01, ***P < 0.001, ****P < 0.0001). **E** Relative qPCR expression of ROS-related genes SOD1 and SOD2 in human C20 cells stimulated with OL-EV or OL-HSPB8-EV either non-activated or TNFα/IL1β activated. As a positive control, the ROS inducer PMA was used. Values are represented as the mean ± SEM of three independent biological replicates. Data were normalized to non-stimulated equals 1. One-way ANOVA was used to determine statistical significance where *P* < 0.05 was considered statistically significant

The biological process by which autophagy levels in cells are regulated following stress conditions involves oxidative stress. Oxidative stress occurs in cells when reactive oxygen species (ROS) generation overwhelms the cells' natural anti-oxidant defenses. Increased ROS and oxidative damage play a vital role in neuroinflammatory diseases [37]. We investigated the role of OL-HSPB8-EVs in modulating oxidative stress due to imbalanced ROS signaling activities in chronic inflammation. To this end, microglial C20 cells and a primary mixed neural culture were challenged with PMA or TNFα/IL1β activated. The ROS signaling activities were detected using the cell-permeable probe, 2'-7'-dichlorodihydrofluorescein diacetate (DCFH-DA). The redox state of a cell was quantified with flow cytometry [38]. Results suggest that stimulation of OL-EVs and OL-HSPB8-EVs showed a non-significant reduction in ROS production in both the non-activated and TNFα/IL1β activated microglial C20 cells (Fig. 4A, B). However, with OL-EVs stimulation, PMA-treated microglial C20 cells report a partial reduction of ROS species, but OL-HSPB8-EVs can significantly reduce the ROS production in PMA-treated cells (Fig. 4A, B), lowering the ROS species level to normal. Furthermore, the impact of OL-HSPB8-EVs on the ROS reduction is markedly confirmed in PMA-treated primary mixed neural culture (Fig. 4C, D). In line with this observation, a qPCR experiment was performed to examine the mRNA expression of copper-zinc superoxide dismutase (SOD1) and manganese superoxide dismutase (SOD2) genes involved in metabolizing superoxide radicals to molecular oxygen and hydrogen peroxide (Fig. 4E). The effects of both EV subsets on ROS endogenous gene activities upon oxidative stress were assessed. Results demonstrate that both SOD1 and SOD2 expression levels were slightly increased in PMA-treated and TNFα/IL1β activated microglial C20 cells. However, no significant effect was observed after EVs stimulation (Fig. 4E).

Collectively, the results suggest that OL-HSPB8-EVs can significantly reduce ROS activities and influence redox signaling in microglia to modulate environmental oxidative stress following chronic inflammation.

### OL-EVs and OL-HSPB8-EVs stimulated cells do not promote cell death in vitro

**Fig. 5:**
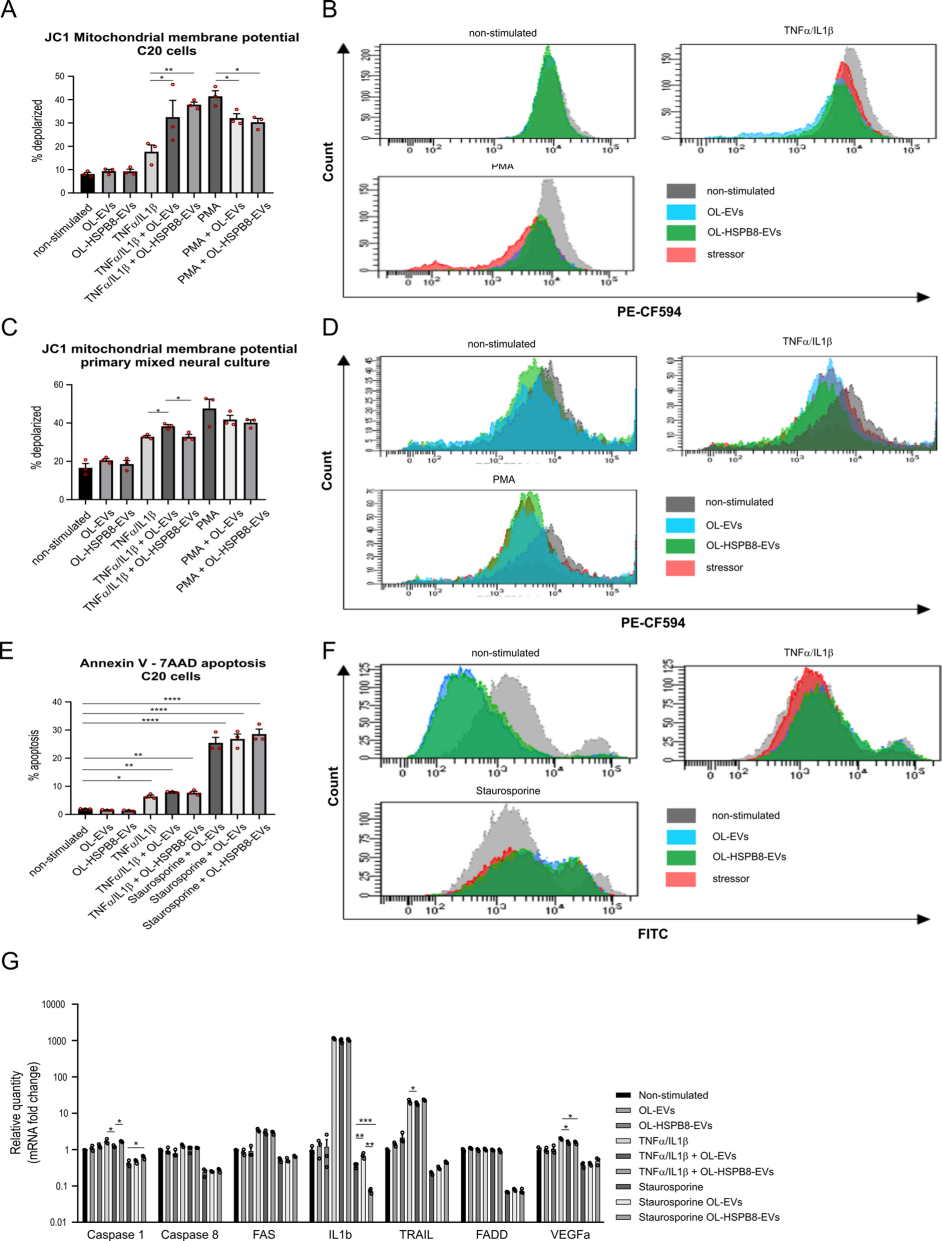
OL-HSPB8-EVs do not promote cell death in C20 cell line and primary mixed neural cultures. **A**–**F** Flow cytometry quantification of early apoptosis by using the JC1 dye to detect the mitochondrial membrane potential (**A**–**D**) and by using Annexin V to measure phosphatidylserine exposure **E**, **F** in the human microglial C20 cell line and a primary mixed neural culture stimulated with OL-EVs or OL-HSPB8-EVs. Cells were either activated with TNFα/IL1β or non-activated. The positive control was stimulated with PMA or staurosporine dependent for each experiment. Data are presented as mean percentage ± SEM (n = 3 biological replicates per group). Graphpad was used to determine significance by using a one-way ANOVA statistical analysis (*P < 0.05, **P < 0.01). **G** Relative qPCR expression of the apoptosis-related genes Caspase1, Caspase8, FAS, IL1β, TRAIL, FADD, and VEGF in human C20 cells stimulated with OL-EVs or OL-HSPB8-EVs either non-activated, or TNFα/IL1β activated. As a positive control, the apoptosis inducer staurosporine was used. Values are represented as the mean ± SEM of three independent biological replicates. Data were normalized to non-stimulated equals 1. One-way ANOVA was used to determine statistical significance were *P* < 0.05 was considered as statistically significant (*P < 0.05, **P < 0.01, ***P < 0.001, ****P < 0.0001)

Next, we sought to determine whether the observed decrease in PMA-induced ROS activity could be seen as a protective response against early cell death. Therefore, changes in the mitochondrial membrane potential (ΔΨm), indicative of early signs of apoptosis, were monitored. Of note, ΔΨm plays a crucial role in mitochondrial homeostasis through selective elimination of dysfunctional mitochondria [39]. The elimination of damaged mitochondria following chronic inflammation is essential for maintaining cellular health and viability. Therefore, the ability of OL-HSPB8-EVs to rapidly eliminate damaged mitochondria was examined [40]. The JC-1 dye was used to study signs of early apoptosis by monitoring mitochondria depolarization in microglial C20 cells stimulated with both EV subsets. Flow cytometry results show no significant effect in non-activated conditions. However, in TNFα/IL1β-activated microglial C20 cells, a significantly increased percentage of mitochondrial depolarized cells was observed following EVs stimulation (Fig. 5A, B). Moreover, we demonstrated that in microglial C20 cells, both OL-EVs and OL-HSPB8-EVs significantly reduced PMA-induced mitochondrial depolarization compared to PMA-only stimulated cells. The same downwards trend was observed in primary mixed neural culture cells following EVs stimulation. Nonetheless, no significant effect was observed for TNFα/IL1β-activated cells stimulated with OL-HSPB8-EV compared to control activated cells (Fig. 5C, D).

Another early sign of apoptosis is the translocation of membrane phosphatidylserine (PS) from the inner side of the plasma membrane to the surface. Therefore, Annexin V, a Ca^2+^-dependent phospholipid-binding protein with a high affinity for PS conjugated to a FITC label, was used to detect PS exposure in EVs stimulated cells by flow cytometry. In line with previous data, our results suggest that microglial C20 cells stimulated with either OL-EVs or OL-HSPB8-EVs did not impair membrane PS exposure compared to non-stimulated cells (Fig. 5E, F). Notably, no significant increase in TNFα/IL1β-activated or staurosporine-treated microglial C20 cells was observed (Fig. 5E). Overall, our results show that both the native and the modified OL-EV subsets do not elicit apoptosis in vitro.

To confirm that OL-HSPB8-EV stimulation in microglia C20 cells does not elicit cell death, a qPCR analysis was performed on genes involved in cellular inflammation, cell death, and survival signaling pathways. Expression levels of IL-1β, caspase 1, caspase 8, FADD, FAS, TNFSF10 (TRAIL), and VEGFA were determined in cells stimulated with OL-EVs and OL-HSPB8-EVs, either in TNFα/IL1β-activated cells, staurosporine-treated cells, or non-activated cells. Overall, a slight reduction or no significant change in gene expression of all the genes tested in non-activated cells stimulated with either OL-EVs and OL-HSPB8-EVs was observed (Fig. 5G). In addition, a slightly significant decrease in caspase 1, TRAIL, and VEGFA gene expression in TNFα/IL1β-activated cells was noted upon OL-EV stimulation. Furthermore, a significant decrease in IL1β expression in cells activated with staurosporine and stimulated with OL-HSPB8-EVs compared to staurosporine-only cells was observed. Taken together, the results show that stimulation of native and modified OL-EVs in cells does not induce major cell death activities in vitro.

## Discussion

Our study reveals that oligodendroglia-derived EVs enriched with HSPB8 modulate microglial and primary mixed neural cells survival communication by sustaining chaperone activity and autophagy and as well as by reducing oxidative stress-induced apoptotic cell death. Specifically, we analyzed the biological effects of in vitro produced small EVs from wild-type native oligodendroglia (OL-EVs) and stable HSPB8-expressing oligodendroglia (OL-HSPB8-EVs) in chronic inflammation. We demonstrate that both EV subsets can be internalized by the C20 recipient microglial cell lines or primary mixed neural culture cells, validating that engineered EVs from oligodendroglia origins can be internalized into different cell types. Additionally, we show that EV uptake is not overtly changed under inflammatory conditions [41]. Furthermore, elucidating the biological functions of the engineered EVs-mediated cell-to-cell communication is a prerequisite for understanding how signaling processes could influence cellular inflammatory responses [42].

We show that OL-EVs and OL-HSPB8-EVs could reduce protein aggregation in stressed conditions, with OL-HSPB8-EVs showing a superior dose-dependent chaperone activity compared to OL-EVs. Our findings support the interpretation that impaired HSPB8 expression levels could render cells more vulnerable to proteotoxic insults, while its induction could protect against the toxicity exerted by the accumulation of misfolded proteins. These results align with Mymrikov et al. previous work showing that additional administration of HSPB8 significantly reduces cytosolic protein aggregation [43]. However, to our knowledge, we provide for the first time evidence that HSPB8 could be enriched within EVs and that the chaperone activity is preserved.

Protecting the functionality of HSPB8 is highly relevant towards the therapeutic potential in neuroinflammatory diseases. For instance, previous studies showed an increased expression of HSPB8 in the white matter of multiple sclerosis (MS) patients, although HSPB8 expression was not detected in microglia and oligodendroglia cells [44]. Additionally, published findings show that administration of exogenous HSPB5 triggered a transient beneficial response against MS lesion activity [45]. Since these HSPBs are directly administered or secreted into the unfavorable inflammatory environment, we reason that their functionality and cytoprotective activity could be compromised and limited. Therefore, packaging HSPB8 within EVs is an example of how chaperones are sustained in the inflammatory environment and maintain their protective activities in response against neuroinflammation.

Next, we demonstrated the presence of BAG3 and LC3B in the protein lysate of both EV subsets, with a slightly increasing trend observed in the OL-HSPB8-EVs protein lysate. Additionally, an increase in LC3B-II protein lysate and autophagic flux was observed on the cellular level in live microglial cells and primary mixed neural culture cells stimulated with either OL-EVs or OL-HSPB8-EVs. Furthermore, upon TNFα/IL1β activation, no further effect of EV stimulation on LC3B-II was observed, possibly due to the ceiling effect of TNFα/IL1β activation. In line with our findings, Rodriguez et al. show that BAG3 regulates the total LC3 protein levels through a translational but not transcriptional mechanism [46]. Moreover, neural stem cell-derived EVs enhanced autophagy by increasing the expression of LC3 and Beclin1, confirming that the contents of EVs could play an important role in the autophagy process [47]. The co-chaperone BAG3 promotes autophagy and inhibits apoptosis by interacting with molecular chaperones and other anti-apoptotic proteins [48]. Published reports show that selective autophagy, including chaperone-assisted selective autophagy (CASA), plays a vital role in neuroinflammation [49]. Interestingly, our findings show that modified EVs contained components of the CASA complex able to recognize misfolded proteins via the HSPB8/BAG3 complex, recruiting the ubiquitin–proteasome system [49]. Of note, modified EVs loaded with HSPB8 showed a superior chaperone activity compared to the native OL-EVs, suggesting that under cellular stress, the modified EVs cargo content including BAG3 together with the molecular chaperones HSPB8 and HSP70 as well as the ubiquitin receptor p62/SQSTM1 can uniquely target aggregation-prone misfolded proteins to autophagic degradation. Therefore, in searching for a novel nanotherapeutic approach to modulate chronic neuroinflammatory responses, OL-HSPB8-EVs is a promising candidate.

Understanding the biological effects of genetically engineered OL-EVs in cells can provide insights into the generation of specific anti-inflammatory nanocarriers to modulate toxic microglial neuroinflammatory responses in chronic demyelinating and neurodegenerative diseases. The progression of many neurodegenerative disorders is characterized by oxidative stress-induced damage to cellular lipids and proteins, resulting in cell death. Here, we revealed that OL-HSPB8-EVs could effectively interfere with PMA induced ROS production by reducing ROS accumulation and activity in microglia C20 cells and primary mixed neural culture cells, thereby modulating oxidative stress following cellular inflammation. PMA treated cells significantly increased ROS production compared to non-treated cells. However, the PMA induced ROS production was countered by stimulating the cells with OL-HSPB8-EVs. This observation points toward the beneficial role of OL-HSPB8-EVs to modulate the imbalance in excess ROS generation. This specific effect of OL-HSPB8-EVs may be of great importance for treating neurodegenerative diseases, in which excessive production and/or insufficient removal of ROS leads to tissue damage. Additionally, the protective effect of OL-HSPB8-EVs is further validated by the small increase in the expression of the cytoplasmic Cu/ZnSOD (SOD1) and the mitochondrial MnSOD (SOD2) upon EV stimulation. Superoxide dismutases are well-known anti-oxidants that catalyze the conversion of O_2_• towards H_2_O_2_, thereby playing a critical role in reducing oxidative stress and mitochondrial dysfunction [50, 51]. Our results are supported by a previous study demonstrating the therapeutic effect of stem cell-derived EVs in reducing oxidative stress [6]. Consequently, given that EVs can cross the blood–brain barrier where they can interact with different cell types, OL-HSPB8-EVs are a promising tool for further development and use in controlling excessive ROS production in therapeutic strategies aiming at engineering and targeting nanocarriers with an efficient anti-oxidant activity.

Additionally, stimulation of PMA treated cells with OL-EVs or OL-HSPB8-EVs reduces the mitochondrial depolarization compared to PMA treated cells only, again verifying the protective effect of OL-HSPB8-EVs against ROS production and subsequently against mitochondrial depolarization [52]. Notably, in cells treated with a combination of TNFα and IL1β, an increased mitochondrial depolarization was observed, which could be explained due to an early release of superoxide, resulting in a release of cytochrome c from the mitochondria into the cytosol, initiating apoptosis [40, 53]. Further TNFα/IL1β activation and stimulation with oligodendroglia EVs subsets pushed cells more towards mitochondrial depolarization, which could target these mitochondria to undergo mitophagy, restoring mitochondrial homeostasis upon cellular stress [54–56].

The effect of both native and modified OL-EVs subsets on apoptosis was further determined by investigating the exposure of phosphatidylserine (PS) at the outer surface of the plasma membrane. No significant results were determined upon EVs stimulation, showing that the OL-EVs subsets do not trigger apoptosis. In addition, treatment of the cells with the apoptosis inducers TNFα/IL1β or staurosporine showed a significant increase in PS exposure. However, no further effect of EV stimulation was observed, confirming that both native and modified OL-EVs in cells do not induce cell death activities in vitro [57–59].

## Conclusions

Our results provide new insights into the development of novel biological HSPB8-nanocarriers as promising therapeutic targets for regulating cellular inflammatory responses. Our findings suggest that in vitro small chaperone-loaded EVs could be further exploited as a new tool to advance cell-type-specific therapeutic approaches controlling neuroinflammatory diseases.

## Supplementary Information

**Additional file 1**. qPCR primer sequences.

**Additional file 2**. OL-EVs and OL-HSPB8-EVs activate autophagy: (A,B) Western blotting analysis of LC3B-II, BAG3, SQSTM1/P62, and HSPB8 in human C20 cells stimulated with OL-EVs or OL-HSPB8-EVs either TNFα/IL1β activated or non-activated. As a positive control, the autophagy inducer rapamycin was used. All conditions were treated with chloroquine to inhibit lysosomal degradation. Protein levels were calculated from at least two independent experiments and normalized to β-actin. Band intensities were determined by quantifying the mean pixel gray values using the ImageJ software. Data are presented as mean ± SEM. Statistical significance was determined by using a one-way ANOVA analysis in Graphpad (*P < 0.05, ****P < 0.0001).

## Data Availability

The datasets used and/or analyzed during the current study are available from the corresponding author upon reasonable request.

## Abbreviations

BAG3: BCL-2–associated anthanogene 3
CNS: Central nervous system
DCFH-DA: 2′-7′-Dichlorodihydrofluorescein diacetate
DMEM: Dulbecco’s Modified Eagle’s Medium
EVs: Extracellular vesicles
FBS: Fetal bovine serum
HSPB8: Small heat-shock protein B8
IL1β: Interleukin 1-beta
KO: Knock-out
MAP1LC3: Microtubule-associated protein 1 light chain
NTA: Nanoparticle tracking analysis
OL: Oligodendroglia
PBS: Phosphate-buffered saline
PMA: Phorbol-12-Myristate-13-Acetate
PS: Phosphatidylserine
qPCR: Quantitative real-time PCR
ROS: Reactive oxygen species
SEC: Size-exclusion chromatography
SOD1: Copper-zinc superoxide dismutase
SOD2: Manganese superoxide dismutase
TEM: Transmission electron microscopy
TNFα: Tumor necrosis factor-alpha
