# Supplementary material for: Oligodendroglia-derived extracellular vesicles activate autophagy via LC3B/BAG3 to protect against oxidative stress with an enhanced effect for HSPB8 enriched vesicles
Source: Cell Commun Signal. 2022 May 5;20:58. doi: 10.1186/s12964-022-00863-x (PMC9069805; doi:10.1186/s12964-022-00863-x)
Supplement: Supplementary file 2 — Additional file 1. qPCR primer sequences. [file 12964_2022_863_MOESM2_ESM.pdf]

Additional file 1: qPCR primer sequences

	Forward primer	Reverse primer
**HSPB8**	AATGTGCACAGCTTCAAGCC	ACAATGCCACCTTCTGCTG
**SOD1**	CTCACTCTCAGGAGACCATTGC	CCACAAGCCAAACGACTTCCAG
**SOD2**	CTGGACAAACCTCAGCCCTAAC	AACCTGAGCCTTGGACACCAAC
**Caspase1**	GCTGTACCCCAGATTTTGTAGCA	TCCAATAATGGACAAGTCAAGCC
**Caspase8**	ATCAATCAGAAGGGAAGACAAGTT	AGTAAGCAACAAGGATGACAAGA
**FAS**	GGACCCAGAATACCAAGTGCAG	GTTGCTGGTGAGTGTGCATTCC
**IL1b**	GGTGCTGATGTACCAGTTGGG	CCAAAGAAGAAGATGGAAAAGC
**TRAIL**	TGGCAACTCCGTCAGCTCGTTA	AGCTGCTACTCTCTGAGGACCT
**FADD**	TCTCCTCTCTGAGACTGCTAA	AGAGAGTGCTGTGTGTCAATC
**VEGFA**	TTGCCTTGCTGCTCTACCTCCA	GATGGCAGTAGCTGCGCTGATA
**GAPDH**	CGGAGTCAACGGATTTGGTCG	AGCCTTCTCCATGGTGGTGAAGAC
**RPL13a**	GCCCTACGACAAGAAAAAGCG	TACTTCCAGCCAACCTCGTGA
