# Supplementary material for: Oligodendroglia-derived extracellular vesicles activate autophagy via LC3B/BAG3 to protect against oxidative stress with an enhanced effect for HSPB8 enriched vesicles
Source: Cell Commun Signal. 2022 May 5;20:58. doi: 10.1186/s12964-022-00863-x (PMC9069805; doi:10.1186/s12964-022-00863-x)
Supplement: Supplementary file 3 — Additional file 2. OL-EVs and OL-HSPB8-EVs activate autophagy: (A,B) Western blotting analysis of LC3B-II, BAG3, SQSTM1/P62, and HSPB8 in human C20 cells stimulated with OL-EVs or OL-HSPB8-EVs either TNFα/IL1β activated or non-activated. As a positive control, the autophagy inducer rapamycin was used. All conditions were treated with chloroquine to inhibit lysosomal degradation. Protein levels were calculated from at least two independent experiments and normalized to β-actin. Band intensities were determined by quantifying the mean pixel gray values using the ImageJ software. Data are presented as mean ± SEM. Statistical significance was determined by using a one-way ANOVA analysis in Graphpad (*P < 0.05, ****P < 0.0001). [file 12964_2022_863_MOESM3_ESM.pdf]

A

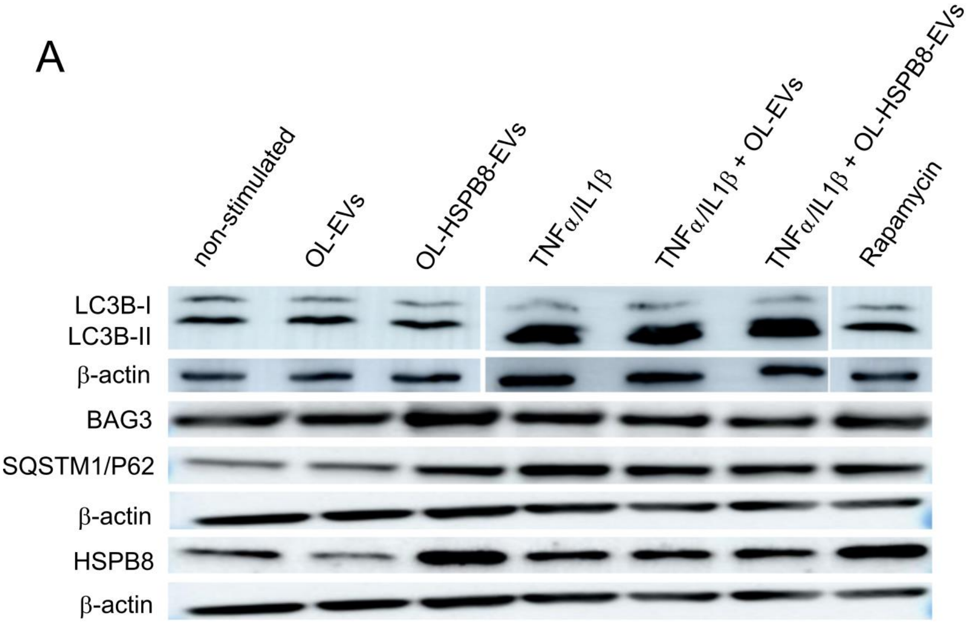

B

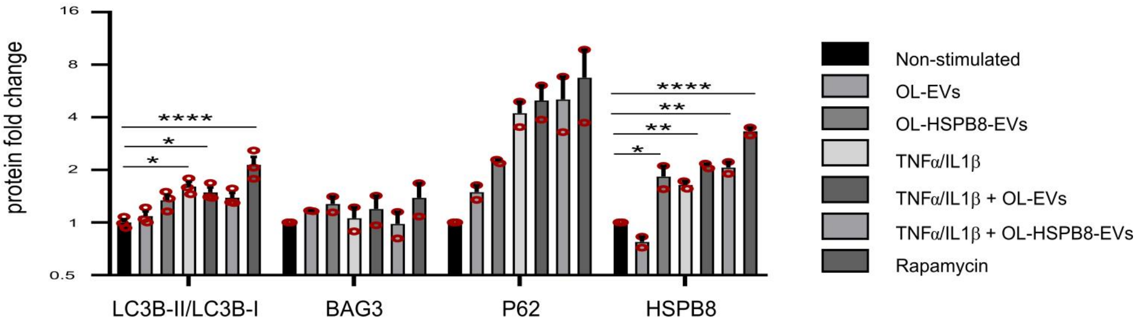

Additional file 2: OL-EVs and HSPB8-EVs activate autophagy. (**A-B**) Western blotting analysis of LC3B-II, BAG3, SQSTM1/P62, and HSPB8 in human C20 cells stimulated with OL-EVs or OL-HSPB8-EVs either TNFα/IL1β activated or non-activated. As a positive control, the autophagy inducer rapamycin was used. All conditions were treated with chloroquine to inhibit lysosomal degradation. Protein levels were calculated from at least two independent experiments and normalized to β-actin. Band intensities were determined by quantifying the mean pixel gray values using the ImageJ software. Data are presented as mean ± SEM. Statistical significance was determined by using a one-way ANOVA analysis in Graphpad (*$P < 0.05$, ****$P < 0.0001$).
